# Targeted sequencing of 36 known or putative colorectal cancer susceptibility genes

**DOI:** 10.1002/mgg3.317

**Published:** 2017-07-23

**Authors:** Melissa S. DeRycke, Shanaka Gunawardena, Jessica R. Balcom, Angela M. Pickart, Lindsey A. Waltman, Amy J. French, Shannon McDonnell, Shaun M. Riska, Zachary C. Fogarty, Melissa C. Larson, Sumit Middha, Bruce W. Eckloff, Yan W. Asmann, Matthew J. Ferber, Robert W. Haile, Steven Gallinger, Mark Clendenning, Christophe Rosty, Aung K. Win, Daniel D. Buchanan, John L. Hopper, Polly A. Newcomb, Loic Le Marchand, Ellen L. Goode, Noralane M. Lindor, Stephen N. Thibodeau

**Affiliations:** ^1^ Department of Laboratory Medicine and Pathology Mayo Clinic Rochester Minnesota; ^2^ Department of Biomedical Statistics and Informatics Mayo Clinic Rochester Minnesota; ^3^ Medical Genome Facility Mayo Clinic Rochester Minnesota; ^4^ Department of Health Sciences Research Mayo Clinic Jacksonville Florida; ^5^ Division of Oncology Department of Medicine Stanford University Stanford California; ^6^ Department of Surgery Mount Sinai Hospital Toronto Ontario Canada; ^7^ Colorectal Oncogenomics Group Genetic Epidemiology Laboratory Department of Pathology The University of Melbourne Parkville Victoria Australia; ^8^ Envoi Specialist Pathologists Herston Queensland Australia; ^9^ School of Medicine University of Queensland Herston Queensland Australia; ^10^ Centre for Epidemiology and Biostatistics Melbourne School of Population and Global Health The University of Melbourne Parkville Victoria Australia; ^11^ Genetic Medicine and Familial Cancer Centre The Royal Melbourne Hospital Parkville Victoria Australia; ^12^ Public Health Sciences Division Fred Hutchinson Cancer Research Center Seattle Washington; ^13^ Epidemiology Program University of Hawaii Cancer Center Honolulu Hawaii; ^14^ Department of Health Sciences Research Mayo Clinic Scottsdale Arkansas

**Keywords:** Colorectal cancer, Familial Colorectal Cancer Type X, germline variants, young onset

## Abstract

**Background:**

Mutations in several genes predispose to colorectal cancer. Genetic testing for hereditary colorectal cancer syndromes was previously limited to single gene tests; thus, only a very limited number of genes were tested, and rarely those infrequently mutated in colorectal cancer. Next‐generation sequencing technologies have made it possible to sequencing panels of genes known and suspected to influence colorectal cancer susceptibility.

**Methods:**

Targeted sequencing of 36 known or putative CRC susceptibility genes was conducted for 1231 CRC cases from five subsets: (1) Familial Colorectal Cancer Type X (*n* = 153); (2) CRC unselected by tumor immunohistochemical or microsatellite stability testing (*n* = 548); (3) young onset (age <50 years) (*n* = 333); (4) proficient mismatch repair (MMR) in cases diagnosed at ≥50 years (*n* = 68); and (5) deficient MMR CRCs with no germline mutations in MLH1, MSH2, MSH6, or PMS2 (*n* = 129). Ninety‐three unaffected controls were also sequenced.

**Results:**

Overall, 29 nonsense, 43 frame‐shift, 13 splice site, six initiator codon variants, one stop codon, 12 exonic deletions, 658 missense, and 17 indels were identified. Missense variants were reviewed by genetic counselors to determine pathogenicity; 13 were pathogenic, 61 were not pathogenic, and 584 were variants of uncertain significance. Overall, we identified 92 cases with pathogenic mutations in *APC*,*MLH1*,*MSH2*,*MSH6*, or multiple pathogenic *MUTYH* mutations (7.5%). Four cases with intact MMR protein expression by immunohistochemistry carried pathogenic MMR mutations.

**Conclusions:**

Results across case subsets may help prioritize genes for inclusion in clinical gene panel tests and underscore the issue of variants of uncertain significance both in well‐characterized genes and those for which limited experience has accumulated.

## Introduction

Colorectal cancer (CRC) is the third most commonly diagnosed cancer for both men and women in the United States with an estimated 132,700 new cases and more than 49,000 deaths in 2015 (Siegel et al. 2015). Approximately 10% of CRC cases are familial, with shared genetic and environmental factors both likely influencing the development of disease (Henrikson et al. 2015). Approximately 5% of CRC cases are considered hereditary, harboring an identified pathogenic single‐gene alteration in genes established to be associated with a substantial increased risk of disease (Burt 2000; Lichtenstein et al. 2000; Chung and Rustgi 2003; Grady 2003; Lynch and de la Chapelle 2003). Several genes have been identified as CRC susceptibility genes, including those implicated in mismatch repair (MMR), responsible for Lynch Syndrome [*MLH1* (OMIM: 120436), *MSH2* (OMIM: 609309), *MSH6* (OMIM: 600678), *PMS2* (OMIM: 600259), and *EPCAM* (OMIM: 185535)]. Several other genes involved in other syndromes predisposing to CRC include *APC* (Familial Adenomatous Polyposis, OMIM: 611731), *MUTYH* (MUTYH‐Associated Polyposis, OMIM: 604933), *TP53* (OMIM: 191170) and *CHEK2* (Li‐Fraumeni syndrome, OMIM: 604373), *STK11* (Peutz‐Jegher syndrome, OMIM: 602216), *PTEN* (Cowden syndrome, OMIM: 601728), *BMPR1A* and *SMAD4* (Juvenile Polyposis syndrome, OMIM: 601299 and 600993, respectively), and *POLD1* and *POLE* (Oligopolyposis, OMIM: 174761 and 174762, respectively) (Liu et al. 2000; Smith et al. 2001; Grady and Markowitz 2002; Suchy et al. 2010; Kastrinos and Syngal 2011; Lubbe et al. 2011; Palles et al. 2013). Additional genes suspected of being involved with CRC susceptibility include those involved in DNA repair [*MLH3* (OMIM: 604395), *MSH3* (OMIM:600887), *NUDT1* (OMIM: 600312), *OGG1* (OMIM: 601982), *PALB2* (OMIM: 610355), *PMS1* (OMIM: 600258), and *RECQL5* (OMIM: 603781)], Transforming Growth Factor Beta 1 signaling [*BMP4* (OMIM: 112262), *SMAD1* (OMIM: 601595), *SMAD2* (OMIM:601366), *SMAD3* (OMIM: 603109), *STK11IP* (OMIM: 607172), *TGFB1* (OMIM: 190180), *TGFBR1* (OMIM: 190181), and *TGFBR2* (OMIM: 190182)], Wnt signaling [*AXIN1* (OMIM: 603816), *AXIN2* (OMIM (604025), *CTNNB1* (OMIM: 116806)], Bloom syndrome (*BLM*, OMIM: 604610), spindle assembly checkpoint (*BUB1*, OMIM: 602452), Birt‐Hogg‐Dube syndrome (*FLCN*, OMIM: 607273), or those with mutations or methylation in CRC or other tumors [*CDH1* (OMIM: 192090), *CDKN1B* (OMIM: 60778), *CDKN2A* (OMIM: 600160), and *GALNT12* (OMIM: 610290)] (Myeroff et al. 1995; Eppert et al. 1996; Ilyas et al. 1997; Shin et al. 2004; Valle et al. 2008; Goto et al. 2009; Guda et al. 2009; Nahorski et al. 2010; Morak et al. 2011; Fleming et al. 2013; Lao et al. 2013; de Voer et al. 2013, 2015; Mazzoni and Fearon 2014).

Currently, several methods are being used to identify mutations in hereditary colorectal cancer (HCC) susceptibility genes. For evaluation of Lynch syndrome in particular, testing algorithms may include microsatellite instability (MSI) and immunohistochemistry (IHC) analysis, tumor and germline hypermethylation, analysis, and germline sequencing and dosage analysis. Universal MSI or IHC testing of CRCs has also been advocated in order to identify individuals with Lynch Syndrome (Giardiello et al. 2014). For the evaluation of other HCC syndromes, genetic testing is typically limited to germline analysis. Previously, genetic testing for HCC syndromes was limited to single gene tests, performed in a cascade fashion when necessary; thus, only a very limited number of genes were tested, and rarely in those infrequently mutated in CRC. As next‐generation sequencing (NGS) technologies advance and costs decrease, sequencing panels of known HCC susceptibility genes are becoming increasingly common. These panels frequently include analysis of candidate CRC risk genes, for which little is known about the spectrum of pathogenic disease‐associated variants. Additionally, in both well‐established and candidate HCC risk genes, many rare variants occur that are not easily classified for pathogenicity (missense, synonymous, intronic, and intergenic variants) and many of these remain categorized as variants of uncertain significance (VUS). Determining the type and frequency of variations in these genes in CRC cases compared to unaffected controls may help in distinguishing pathogenic and benign variants and may help prioritize testing for family members of affected individuals.

In this study, we screened for germline mutations in 36 genes across five categories of CRC cases, including (1) Familial Colorectal Cancer Type X (FCCTX) which meet the Amsterdam Criteria I for Lynch Syndrome, but have normal mismatch repair function (microsatellite stable [MSS] and/or normal expression of four MMR proteins encoded by *MLH1*,* MSH2*,* MSH6*, and *PMS2* by IHC) in the tumor (Lindor et al. 2005a), (2) unselected CRC cases with no prior IHC or MSI testing completed, (3) proficient MMR (pMMR) or unknown MMR status cases diagnosed ≤50 years (diagnosed <50 years of age), (4) proficient MMR (pMMR) cases, based on MSI or IHC testing, diagnosed ≥50 years, and (5) deficient MMR (dMMR) cases where no germline mutation has been previously identified by sequencing or multiplex ligation probe assay (MLPA) in the four main MMR genes (Lindor et al. 2005b; Boland and Goel 2010).

## Materials and Methods

### Ethics compliance

All participants provided informed consent. Protocols were approved by the Ontario Cancer Research Ethics Board, University of Southern California Institutional Review Board, University of Melbourne Human Research Ethics Committee, University of Hawaii Institutional Review Board, Mayo Clinic Institutional Review Board, and Fred Hutchinson Cancer Research Center Institutional Review Board.

### Subjects

Subjects were selected from the Colon Cancer Family Registry (Colon CFR) for mutation screening as part of the overall genetic characterization of this registry. The Colon CFR is an NCI‐supported consortium established to create an infrastructure for interdisciplinary studies of the genetic and molecular epidemiology of CRC (Newcomb et al. 2007). Families were enrolled between 1998 and 2012 as part of the following registries: Australasian Colorectal Cancer Family Registry (Melbourne, Victoria, Australia), University of Hawaii Colorectal Cancer Family Registry (Honolulu, Hawaii), Mayo Clinic Cooperative Family Registry for Colon Cancer Studies (Rochester, MN), Ontario Familial Colorectal Cancer Registry (Toronto, Ontario, Canada), Seattle Colorectal Cancer Family Registry (Seattle, Washington), and University of Southern California Consortium (USCC) Colorectal Cancer Family Registry (Los Angeles, California).

Risk factor data, blood samples, and pathology reports were collected from participants using standardized protocols, and germline DNA was isolated from blood. Population‐ and clinic‐based individuals chosen for germline DNA sequencing were divided into five case groups (persons with CRC), namely: (1) Familial Colorectal Cancer Type X cases, which meet the Amsterdam Criteria I for Lynch Syndrome (Vasen et al. 1991), but have normal mismatch repair function (microsatellite stable [MSS] and/or normal expression of four MMR proteins encoded by *MLH1*,* MSH2*,* MSH6*, and *PMS2* by IHC) in the tumor (“FCCTX”; *n* = 153); (2) CRC cases with no prior IHC or MSI testing (“unselected”; *n* = 548); (3) proficient MMR (pMMR) or unknown MMR status cases diagnosed ≤50 years (“young onset”; *n* = 333); (4) cases diagnosed ≥50 years with proficient DNA mismatch repair based on MSI or IHC testing (“pMMR”; *n* = 68); and (5) cases with deficient MMR in tumor but with no germline mutation identified in the gene with lost protein expression by sequencing or multiple ligation probe assay (MLPA) (“dMMR”; *n* = 129). Several samples could be classified into more than one case group; particularly high overlap was present in the FCCTX, young onset, and pMMR cases. For clarity in reporting, samples were only included in a single group, with priority for classification proceeding FCCTX > young onset > pMMR. In addition, we chose a sample of 93 persons without CRC from among the spouses of cases as “controls”.

### Custom capture gene selection

Thirty‐six genes known [*APC* (NM_000038.5), *AXIN2* (NM_004655.3), *BMP4* (NM_001202.3), *BMPR1A* (NM_004329.2), *CDH1* (NM_004360.3), *CHEK2* (NM_007194.3), *MLH1* (NM_000249.3), *MSH2* (NM_000251.2), *MSH3* (NM_002439.4), *MSH6* (NM_000179.2), *MUTYH* (NM_001128425.1), *PMS1*(NM_000534.4), *PTEN* (NM_000314.4), *SMAD4* (NM_005359.5), *STK11* (NM_000455.4), *STK11IP* (XM_005246262.1), *TGFBR2* (NM_001024847.2), and *TP53* (NM_000546.5)] or suspected [*AXIN1* (NM_181050.2 *), BLM* (NM_000057.2), *BUB1* (NM_004336.4), *CDKN1B* (NM_004064.3), *CDKN2A* (NM_000077.4), *CTNNB1* (NM_001904.3), *FLCN (* NM_144997.5), *GALNT12* (NM_024642.4), *MLH3* (NM_001040108.1), *NUDT1* (NM_002452.3), *OGG1* (NM_002542.5), *PALB2* (NM_024675.3), *RECQL5* (NM_004259.6), *SMAD1* (NM_005900.2), *SMAD2* (NM_001003652.3), *SMAD3* (NM_005902.3), *TGFB1* (NM_000660.4), and *TGFBR1* (NM_004612.2)] to be associated with CRC susceptibility were selected for targeted sequencing using Agilent's Custom Capture Kit. All exons and ±30 bp of each exon/intron boundary of each gene were specifically targeted for capture and sequencing.

### Custom capture and sequencing

Paired‐end indexed libraries were prepared using the Agilent Bravo liquid handler following the manufacturer's protocol (Agilent). Briefly, 3 *μ*g of target DNA in 120 *μ*L TE buffer was fragmented using the Covaris E210 sonicator. The settings of duty cycle 10%, intensity 5, cycles 200, time 360 sec generated double‐stranded DNA fragments with blunt or sticky ends with a fragment size mode of between 150–200 bp. The ends were repaired and phosphorylated using Klenow, T4 polymerase, and T4 polynucleotide kinase, after which an “A” base was added to the 3′ ends of double‐stranded DNA using Klenow exo (3′ to 5′ exo minus). Paired‐end Index DNA adaptors (Agilent) with a single “T” base overhang at the 3′ end were ligated and the resulting constructs were purified using AMPure SPRI beads from Agencourt. The adapter‐modified DNA fragments were enriched by four cycles of PCR using SureSelect forward and SureSelect Pre‐Capture Indexing reverse (Agilent) primers. The concentration and size distribution of the libraries were determined using an Agilent Bioanalyzer DNA 1000 chip.

Custom capture of 3.69 Mb was carried out using the Agilent Bravo liquid handler following the protocol for Agilent's SureSelect XT, such that 750 ng of the prepped library was incubated with whole‐exon biotinylated RNA capture baits supplied in the kit for 24 h at 65°C. The captured DNA:RNA hybrids were recovered using Dynabeads MyOne Streptavidin T1 from Dynal. The DNA was eluted from the beads and purified using Ampure XP beads from Agencourt. The purified capture products were then amplified using the SureSelect Post‐Capture Indexing forward and Index PCR reverse primers (Agilent) for 12 cycles. Libraries were validated and quantified on the Agilent Bioanalyzer.

Libraries were pooled at equimolar concentrations in batches of 96 samples and loaded onto paired‐end flow cells at concentrations of 7–8 pM to generate cluster densities of 600,000–800,000/mm^2^ following Illumina's standard protocol using the Illumina cBot and HiSeq Paired‐end cluster kit version 3. Each pool of samples was run on five lanes of a flow cell to generate a minimum of 200x coverage per sample.

The flow cells were sequenced as 101 bp X 2 paired‐end reads on an Illumina HiSeq 2000 using TruSeq SBS sequencing kit version 3 and HiSeq data collection version 1.4.8 software. Base calling was performed using Illumina's RTA version 1.12.4.2.

### Subject and variant filtering

Six subjects were excluded due to poor coverage at either 10× (<95%) or 40× (<60%) or low concordance (<95%) with the 96 SNP genotyping panel.

Variant files were imported into Golden Helix SVS (8.4.0) and filtered to exclude variants with low read depth (<20), low genotype quality (<30), or a public minor allele frequency (MAF) of ≥0.05 in any ethnic population present in 1000Genomes, ESP, or ExAC.(Exome Variant Server, NHLBI GO Exome Sequencing Project (ESP); Lek et al. 2016; The Genomes Project, 2015).

Variants most likely to disrupt protein expression or function [nonsense variants, frame‐shift insertion/deletion/duplication variants, splice site variants (±2 bases from the exon–intron boundary), initiator codon variants, and stop‐codon variants] were designated as Tier 1 variants. Missense variants and in‐frame insertion/deletion/duplication variants were designated as Tier 2 variants and were evaluated as described below to determine classification as either pathogenic, benign, or VUS. Synonymous variants and those located in introns, untranslated regions, or intergenic regions were excluded from this study.

### Detection of large exonic deletions

All copy number variations (CNVs) were called using an updated version of PatternCNV, which uses all the samples to learn the pattern and variance of the coverage to better enable CNV calling (Wang et al. 2014). It computes the differences in observed coverage versus the common pattern, while penalizing regions associated with larger variability using a weighting scheme. Results from probe‐level CNV are summarized using circular binary segmentation. Further CNV segmentation results were evaluated in three genes, MLH1, MSH2, and MSH6 using −0.5 and 0.5 as log2 ratio cutoff for deletion and amplification, respectively.

### Missense variant review

Review and classification of missense variants were completed by three genetic counselors (authors JRB, AMP, and LAW) as outlined in Figure S1. Variants with a MAF ≥2% and <5% were considered benign. The remaining variants were assessed for available annotation information from a number of sources: Mayo's clinical Molecular Genetics laboratory, InSiGHT database (International Society for Gastrointestinal Hereditary Tumors) for MMR genes, ClinVar mutation database, and the Human Gene Mutation Database (HGMD) (Cooper et al. 1998; Ou et al. 2008; Landrum et al. 2016). Variants that had not been annotated by any of these groups were classified as VUS. For missense variants that had been annotated by InSiGHT, the InSiGHT classification was assigned. Variants in genes tested at Mayo's clinical Molecular Genetic laboratory were assessed for prior experience with Mayo's clinical laboratory. For variants that had been recently annotated (since 2015) per Mayo's clinical laboratory internal databases, the current Mayo classifications were assigned. Of note, for those variants annotated in both the internal Mayo databases and InSiGHT, the InSiGHT classification was used. For variants that had been most recently annotated by Mayo's clinical laboratory prior to 2015, additional review was performed as described below. For variants annotated in ClinVar with a ≥ 2 star rating (requiring multiple submitters and no conflicting interpretations) the ClinVar classification was assigned (Landrum et al. 2016). For variants that had been annotated by Mayo's clinical Molecular Genetics laboratory prior to 2015, those that were annotated in ClinVar but had a < 2 star rating, and those that were annotated only in HGMD, final classification was assigned based on available annotation data incorporated with additional genetic counselor variant review. This review process included assessment of available database annotations, literature review, and analysis using in silico predictive tools. Classifications were determined based on ACMG guidelines (Richards et al. 2015).

### REVEL scores for missense variants

Rare Exome Variant Ensemble Learner (REVEL) is a new ensemble method developed to help predict the pathogenicity of rare missense variants, such as those commonly identified using modern sequencing technologies. The REVEL random forest was trained on recently discovered disease and rare neutral variants, and incorporates scores from multiple individual tools, including: MutPred, VEST, FATHMM, Polyphen, SIFT, PROVEAN, MutationAssessor, MutationTaster, LRT, GERP, SiPhy, phyloP, and phastCons. REVEL scores ranging from 0 to 1 were generated for all missense variants in our study, and were utilized to help determine their relative likelihood of pathogenicity. We chose a threshold of ≥0.5 to be considered likely damaging, corresponding to 75.4% sensitivity and 89.1% specificity (Ioannidis, et al.).

### LSDB submission of identified variants

All variants reported in the article have been submitted to the corresponding Locus Specific Mutation Database (LSDB, http://grenada.lumc.nl/LSDB_list/lsdbs/).

## Results

### Subject characteristics

After exclusions due to poor coverage or concordance (*n* = 6, 0.5%), a total of 1324 individuals were included in the study, including 1231 cases with CRC and 93 controls (Table 1). The majority of cases were Caucasian (76%), while the remaining were African American (11%), Asian (3%), or mixed (10%).

**Table 1:** Sample characteristics

	Australiasia	Ontario	Hawaii	Mayo Clinic	Seattle	USCC	ALL
Age at diagnosis of cases (range)	44.3 (18–73)	48.8 (20–82)	47.4 (27–81)	42.5 (18–90)	42.9 (21–73)	48.6 (16–74)	46.1 (16–90)
Age of controls (range)	47.1 (24–60)	57.0 (35–70)	0	48.4 (27–76)	0	0	49.6 (24–76)
Ethnicity							
Caucasian	173	204	10	269	112	163	931
African American	0	0	0	1	4	134	139
Asian	3	6	20	0	5	10	44
Admix	4	7	6	3	15	82	117
Ascertainment (Cases only)							
Population	113	203	36	126	136	294	908
Clinic	67	14	0	147	0	95	323
Sample group							
FCCTX	35	44	7	37	6	24	153
Unselected	65	120	4	0	68	291	548
Young Onset	36	43	8	159	48	39	333
pMMR	11	9	5	22	4	17	68
dMMR	33	1	12	55	10	18	129
Negative control	19	16	0	58	0	0	93

### Tier 1 variants

Eight percent (*n* = 103) of cases harbored a Tier 1 (nonsense, frame‐shift, splice site, initial codon, and stop loss) variant. The majority carried a single Tier 1 variant, while two unselected cases harbored two Tier 1 variants in different genes, *MLH1* (p.Met35fs) and *MSH6* (p.Lys1358fs), while another case harbored both *APC* (p.His2045fs) and *MSH3* (p.Gln74fs).

Ninety‐two unique Tier 1 variants were identified in 101 individuals, with *MSH6*,* MSH2*,* MLH1*, and *APC* the most frequently mutated genes (20, 13, 13, and 12 unique mutations each, respectively, Tables 2 and S1). Of the 92 variants, four were present only in controls, one was present in both cases and controls, and the remaining 87 were present only in cases. The vast majority of variants were identified in only one individual (*n* = 84); the remaining eight variants were identified in two to five individuals. Individual variant results, for both the entire cohort as well as ethnic subgroups, are shown in Table S1.

**Table 2:** Tier 1 variant overview

Gene	Controls (*n* = 93)					FCCTX (*n* = 153)					Unselected (*N* = 548)				
	Nonsense	FrameShift	Splice site	Initial codon & stop loss	% Carriers	Nonsense	FrameShift	Splice site	Initial codon & stop loss	% Carriers	Nonsense	FrameShift	Splice site	Initial codon & stop loss	% Carriers
APC											3 (3)^b^^**,**^ c	2 (2)			0.9
AXIN1												1 (1)^a^			0.2
AXIN2															
BLM		1 (1)			1.1							1 (1)			0.2
BMP4			1 (1)		1.1										
BMPR1A															
BUB1							1 (1)			0.7		1 (1)^c^			0.2
CDH1															
CDKN1B															
CDKN2A															
CHEK2		1 (1)			1.1	1 (1)	1 (1)			1.3		1 (1)	1 (1)^a^		0.4
CTNNB1															
FLCN											1 (1)		1 (1)	1 (1)	0.5
GALNT12															
MLH1											3 (4)^c^	3 (3)^b^	2 (3)^c^	1 (1)^c^	2.0
MLH3															
MSH2						1 (1)				0.7	4 (5)^a^	2 (2)	2 (2)^a^		1.6
MSH3											1 (1)	1 (1)			0.4
MSH6	1 (1)				1.1		3 (3)			2.0	3 (3)^a^	8 (10)^a^^**,**^ b			2.4
MUTYH						1 (1)				0.7			1 (1)^b^		0.2
NUDT1							1 (1)		1 (1)^b^	1.3					
OGG1														1 (1)	0.2
PALB2															
PMS1											1 (1)^c^	2 (2)			0.5
PTEN															
RECQL5													1 (3)		0.5
SMAD1															
SMAD2															
SMAD3														1 (1)^c^	0.2
SMAD4															
STK11															
STK11IP						1 (1)				0.7	1 (1)	1 (1)^a^			0.4
TGFB1															
TGFBR1	1 (1)				1.1										
TGFBR2															
TP53														1 (1)	0.2
Total	2 (2)	2 (2)	1 (1)		5 (5) 5.4	4 (4)	6 (6)		1 (1)	11 (11) 7.2	17 (19)	23 (25)	8 (10)	5 (5)	53 (59) 10.8

Gene	Young onset (*n* = 333)					pMMR (*n* = 68)					dMMR (*n* = 129)				
	Nonsense	FrameShift	splice site	Initial codon & stop loss	% Carriers	Nonsense	FrameShift	Splice site	Initial codon & stop loss	% Carriers	Nonsense	FrameShift	Splice site	initial codon & stop loss	% Carriers
APC	4 (4)	3 (3)^a^			2.1										
AXIN1															
AXIN2															
BLM		1 (1)	1 (1)		0.6										
BMP4															
BMPR1A		1 (1)^a^			0.3										
BUB1															
CDH1															
CDKN1B															
CDKN2A															
CHEK2		1 (1)			0.3										
CTNNB1															
FLCN															
GALNT12															
MLH1	1 (1)	2 (2)	2 (3)^c^		1.8			1 (1)^a^		1.5					
MLH3															
MSH2	1 (1)	1 (1)	2 (2)		1.2									1 (1)	0.8
MSH3															
MSH6	2 (2)	4 (4)			1.8		1 (1)^b^			1.5		1 (1)			0.8
MUTYH		1 (1)			0.3										
NUDT1															
OGG1															
PALB2															
PMS1															
PTEN															
RECQL5			1 (1)		0.3										
SMAD1															
SMAD2															
SMAD3															
SMAD4		1 (1)			0.3										
STK11															
STK11IP															
TGFB1											1 (1)^b^				0.8
TGFBR1															
TGFBR2															
TP53															
Total	8 (8)	15 (15)	6 (7)		29 (30) 9.0		1 (1)	1 (1)		2 (2) 2.9	1 (1)	1 (1)		1 (1)	3 (3) 2.3

# (#): Number of unique variants (number of individuals with one of the variants).

a Variant is present in African American samples.

b Variant is present in Asian samples.

c Variant is present in Admix samples.

Five Tier 1 mutations were identified in the controls (Table 2) including an *MSH6* nonsense variant (p.Arg1005*) that was confirmed by Sanger sequencing, a nonsense variant in *TGFBR1*, frame‐shift variants in *BLM* and *CHEK2*, and a splice‐site variant in *BMP4*.

The unselected cases had the highest frequency of Tier 1 variants overall (11%, Table 2), as it was both the largest sample group and because neither tumor triage nor mutation screening was performed prior to study inclusion. Thirteen unselected cases (2%) carried a Tier 1 variant in *MSH6*, while 11 (2%), nine (2%), and five (1%) carried a Tier 1 mutation in *MLH1*,* MSH2*, and *APC*, respectively.

Variants were also found frequently in the young onset and FCCTX cases (9% and 7%, respectively; Table 2). *APC* mutations were the most common in the young onset cases (2%), but none were present in the FCCTX cases. Surprisingly, we identified four FCCTX cases with damaging MMR mutations, including one individual with an Arg711* mutation in *MSH2*, and three individuals with frame‐shift mutations in *MSH6* (p.Phe1037fs, p.Ala1320fs, and p.Phe1088fs) (Table S1). All four had IHC results indicating the protein expression of interest was present and normal. MSI testing was performed on two of the subjects’ tumors and indicated microsatellite stable tumors; MSI testing was not completed on the other two. Thus, the available tumor data on these cases would not have led to triage for sequencing for MMR gene mutations. Of the 69 young onset cases without any prior IHC or MSI tumor testing, 16 harbored mutations in MMR genes (23%). Because of the lack of previous MSI or IHC testing and the early onset of disease in these cases, we expected several cases to have pathogenic MMR gene mutations.

Three Tier 1 variants were identified in the dMMR cases, one each in *MSH2*,* MSH6*, and *TGFB1*. The initiator codon of *MSH2* was mutated in one case (c.1A>C), however, previous IHC indicated that MSH2 was present and normally expressed and this variant is classified as a VUS by InSiGHT. MLH1 expression was heterogeneous and PMS2 expression was lost and the tumor was also negative for MLH1 methylation. In the case with the *MSH6* mutation (p.Phe1088fs), IHC studies indicated loss of MLH1 but normal expression of MSH6.

Nearly half of the genes tested had no Tier 1 variants (*AXIN2*,* CDH1*,* CDKN1B*,* CDKN2A*,* CTNNB1*,* GALNT12*,* MLH3*,* PALB2*,* PTEN*,* SMAD1*,* SMAD2*,* STK11*, and *TGFBR2*) and an additional 10 only had a single Tier 1 variant (*AXIN1*,* BMP4*,* BMPR1A*,* OGG1*,* RECQL5*,* SMAD3*,* SMAD4*,* TGFB1*,* TGFBR1*, and *TP53*).

### Large exonic deletions

Twelve cases were identified with a large exonic deletions in MMR genes (MLH1, MSH2, or MSH6 (Table 3). Six large deletions were identified in MLH1, while there were five in MSH2 and two in MSH6. One Unselected case had large deletions in both MLH1 and MSH2. Unselected cases had the largest proportion of large deletions (*n* = 8), with one case each of dMMR, FCCTX, pMMR, and YO harboring a large deletion. Cases with large deletions were diagnosed young, with a median age of diagnosis of 46 (range: 24–60).

**Table 3:** Cases with large exonic deletions

Individual	Sample Category	Gene	Deletion Boundaries	Exons/Intron Deleted	Age Dx	IHC MLH1	IHC MSH2	IHC MSH6
s_1204002002	dMMR	MSH2	chr2:47697669:47708724	intron 10 – intron 15	40	Normal	Failed	Missing
s_1204003924	Unselected	MLH1	chr3:37087820:37092640	Intron 15 – 3′	25	ND	ND	ND
s_1207501563	Unselected	MSH2	chr2:47629431:47649608	5′ – intron 6	49	ND	ND	ND
s_1207501567	Unselected	MLH1	chr3:37084259:37093565	Intron 15 – 3′	60	ND	ND	ND
s_1208804910	Unselected	MLH1	chr3:37061440:42236604	Intron 10 – 3′	57	ND	ND	ND
**s_1208804959**	Unselected	MLH1	chr3:37029085:197686994	5′ – 3′	59	ND	ND	ND
**s_1208804959**	Unselected	MSH2	chr2:47643733:48022675	intron 6 – 3′	59	ND	ND	ND
s_1208804977	Unselected	MSH6	chr2:48023417:55214950	Intron 3 – 3′	51	Normal	Normal	Normal
s_1208805044	Unselected	MLH1	chr3:37083176:37084778	Intron 14 – Intron 15	24	ND	ND	ND
s_1208903221	Unselected	MSH6	chr2:48005252:48034082	5′ – 3′	56	ND	ND	ND
s_1210304581	pMMR Link	MSH2	chr2:47676087:47696198	intron 8 – intron 10	52	ND	ND	ND
s_1210304633	YO	MLH1	chr3:37039963:37051213	Intron 2 – Intron 6	35	ND	ND	ND
s_1211600397	FCCTX	MSH2	chr2:47626940:48023946	5′ – 3′	48	Normal	Normal	Normal

Bold case has two large deletions in different genes.

### Tier 2 variants

A total of 658 missense and 17 in‐frame indels were classified as Tier 2 variants, with 32 being found exclusively in controls. Most Tier 2 variants were present in one to three individuals, concordant with their low minor allele frequency in the public databases. After review of pathogenicity as outlined in the methods, 13 were considered to be pathogenic or likely pathogenic, 61 were considered not pathogenic, likely not pathogenic, or polymorphisms, and the remaining 584 were classified as variants of uncertain significance (VUS) (Table S2). Of the variants only found in controls, one was classified as likely not pathogenic, while the remaining 31 were classified as VUS. Because of the large number of variants classified as VUS in both cases and controls, we also utilized variant REVEL scores to assess pathogenicity, as described in the methods. For variants classified as VUS, we used a REVEL score of >0.5 to be likely damaging, corresponding to 75.4 sensitivity and 89.1 specificity (Ioannidis et al.). Overall, 25% of the VUS missense variants were considered likely damaging using this cutoff (*n* = 144) and these variants, as well as the ones classified as pathogenic or likely pathogenic, are discussed further below (Tables 4 and S2).

**Table 4:** Tier 2 variant overview

Gene	Negative controls (*n* = 93)				FCCTX (*n* = 153)				Unselected (*n* = 548)			
	In‐frame indels	Missense	REVEL > 0.5	% Carriers REVEL > 0.5	In‐frame indels	Missense	REVEL > 0.5	% Carriers REVEL > 0.5	In‐frame indels	Missense	REVEL > 0.5	% Carriers REVEL > 0.5
APC	b	7 (19)^d^	1 (2)	2.2		12 (17)^e^	3 (3)	2.0	2 (7)	27 (62)^c^^,^ d ^,^ e	5 (6)^c^^,^ d	1.1
AXIN1		4 (11)	1 (1)	1.1		5 (9)			1 (1)	8 (36)^c^^,^ d ^,^ e	1 (1)^c^	0.2
AXIN2		2 (2)				5 (5)^d^			1 (2)	15 (44)^c^^,^ e	1 (1)^e^	0.2
BLM		4 (4)				7 (8)^e^			2 (2)	25 (44)^c^^,^ d ^,^ e	2 (2)^c^^,^ e	0.4
BMP4						1 (1)^d^	1 (1)^d^	0.7		6 (11)^c^^,^ e	1 (1)	0.2
BMPR1A		1 (1)								3 (3)^c^^,^ e	1 (1)^e^	0.2
BUB1		2 (2)				4 (5)				9 (11)^c^^,^ e		
CDH1		2 (2)				4 (5)				11 (16)^c^^,^ e	4 (6)^c^^,^ e	1.1
CDKN1B		2 (2)								4 (6)^c^^,^ e		
CDKN2A		2 (8)				1 (12)^b^				5 (34)^c^^,^ e	1 (1)^c^^,^ e	0.2
CHEK2		1 (1)	1 (1)	1.1		2 (2)	1 (1)	0.7	1 (1)	17 (32)^b^^,^ c ^,^ e	8(12)^b^^,^ c	2.2
CTNNB1						2 (2)				4 (4)^e^		
FLCN						2 (2)	1 (1)	0.7		7 (7)^d^^,^ e		
GALNT12		3 (8)	1 (1)	1.1		3 (4)^e^	2 (2)^e^	1.3		8 (16)^b^^,^ c ^,^ d	2 (2)^c^	0.4
MLH1		1 (1)	1 (1)	1.1		4 (4)^c^	2 (2)	1.3		10 (27)^c^^,^ d ^,^ e	5 (5)^d^	0.9
MLH3		6 (14)	1 (1)	1.1		8 (25)^d^^,^ e				24 (119)^c^^,^ d ^,^ e	4 (4)^c^^,^ d ^,^ e	0.7
MSH2		2 (4)				1 (8)^e^			2 (2)	16 (33)^c^^,^ d ^,^ e	11 (13)^c^^,^ d ^,^ e	2.4
MSH3		1 (1)				3 (3)^d^				23 (57)^c^^,^ e	7 (18)^c^^,^ e	3.3
MSH6		4 (5)			1 (1)	2 (2)				15 (31)^c^^,^ e	3 (3)^c^^,^ e	0.5
MUTYH		4 (7)	2 (3)	3.2	1 (1)	9 (14)^b^	6 (8)^a^	5.2		11 (48)^c^^,^ e	5 (19)^c^^,^ e	0.2
NUDT1		2 (2)^d^				2 (4)^c^				3 (18)^c^^,^ d ^,^ e		
OGG1		3 (4)	1 (1)	1.1		3 (3)				9 (15)^e^	3 (7)	1.3
PALB2		7 (13)				11 (41)^c^^,^ d				26 (103)^c^^,^ d ^,^ e		
PMS1		5 (7)	2 (2)	2.2		5 (7)	3 (3)	2.0	1 (1)	18 (85)^b^^,^ c ^,^ e	5 (21)^b^^,^ c ^,^ e	3.8
PTEN						1 (1)	1 (1)	0.7				
RECQL5		6 (7)				14 (23)^c^^,^ e	1 (2)	1.3		25 (83)^b^^,^ c ^,^ d ^,^ e	3 (5)	0.9
SMAD1		2 (2)	1 (1)	1.1		1 (1)				1 (1)	1 (1)	0.2
SMAD2												
SMAD3						1 (1)				1 (1)	1 (1)	0.2
SMAD4		1 (2)	1 (2)	2.2		1 (1)^c^	1 (1)^c^	0.7		2 (2)^e^		
STK11		1 (4)^d^				2 (3)				4 (10)^c^^,^ d ^,^ e		
STK11IP		3 (3)				8 (9)^d^			1 (2)	22 (38)^c^^,^ d ^,^ e		
TGFB1		2 (6)	1 (1)	1.1		2 (7)				4 (28)^e^		
TGFBR1		1 (1)								1 (1)	1 (1)	0.2
TGFBR2		1 (1)				2 (2)				8 (11)^c^^,^ d ^,^ e	1 (1)	0.2
TP53										4 (10)^c^^,^ e	2 (2)^c^	0.4
Total	0	82 (144)	12 (17)	18.3	2 (2)	129 (231)	21 (24)	15.7	11 (18)	376 (1,047)	79 (141)	25.7

Gene	Young onset (*n* = 333)				pMMR (*n* = 68)				dMMR (*n* = 129)			
	In‐frame indels	Missense	REVEL > 0.5	% Carriers REVEL > 0.5	In‐frame indels	Missense	REVEL > 0.5	% Carriers REVEL > 0.5	In‐frame indels	Missense	REVEL > 0.5	% Carriers REVEL > 0.5
APC		23 (48)^d^	4 (4)	1.2	1 (1)	4 (6)^d^^,^ e				9 (16)^c^^,^ d ^,^ e	1 (1)	0.8
AXIN1		7 (21)				4 (9)				5 (15)		
AXIN2		9 (17)^d^^,^ e			1 (1)	6 (7)^d^^,^ e				5 (7)		
BLM	1 (1)	11 (16)^b^^,^ c				4 (4)^c^^,^ d				5 (6)		
BMP4		5 (5)^c^								1 (1)	1 (1)	0.8
BMPR1A		2 (2)	1 (1)	0.3								
BUB1		6 (10)	1 (1)	0.3								
CDH1		2 (6)	1 (3)	0.9		2 (3)^d^	1 (1)^d^	1.5		3 (5)^d^	1 (1)	0.8
CDKN1B	c	2 (2)^c^	1 (1)	0.3						1 (1)		
CDKN2A	d	3 (20)^c^				1 (6)^e^				1 (9)^d^		
CHEK2	e	10 (10)^c^^,^ e	5 (5)	1.5						3 (4)^c^	1 (2)	1.6
CTNNB1	c ^,^ d	1 (1)								1 (1)		
FLCN	c ^,^ e	3 (8)	1 (2)	0.6		3 (3)	2 (2)	2.9		1 (2)^e^		
GALNT12	c ^,^ d ^,^ e	2 (10)^e^								1 (3)		
MLH1	d ^,^ e	14 (19)^c^	8 (9)	2.7		1 (1)^d^				2 (2)	1 (1)	0.8
MLH3		14 (65)^d^^,^ e	2 (2)^d^	0.6		7 (11)^d^				8 (16)^d^^,^ e		
MSH2		8 (20)^e^	4 (4)^e^	1.2		2 (3)^d^			1 (1)	3 (4)	1 (1)	0.8
MSH3		10 (15)^c^^,^ d ^,^ e	5 (9)^e^	2.7		1 (2)	1 (2)	2.9		3 (3)^d^	2 (2)^d^	1.6
MSH6		8 (10)^d^	5 (5)^d^	1.5		1 (1)^e^				8 (10)^d^	1 (1)	0.8
MUTYH		9 (27)^b^^,^ c	5 (16)^b^	4.8		3 (4)^c^^,^ e	1 (1)	1.5		5 (5)^d^	3 (3)	2.3
NUDT1		3 (6)^d^				2 (8)^c^				1 (5)^d^^,^ e		
OGG1		3 (9)	1 (4)	1.2		1 (2)^d^	1 (2)	2.9		5 (6)	2 (2)	1.6
PALB2		16 (58)^c^^,^ d				7 (10)^b^^,^ c ^,^ d				13 (36)^d^		
PMS1		11 (17)^c^	3 (3)^c^	0.9		5 (6)^c^	3 (4)^c^	5.9		8 (10)^e^	2 (2)^e^	1.6
PTEN		1 (1)	1 (1)	0.3								
RECQL5		10 (36)	1 (6)	1.8		5 (5)	1 (1)	1.5	1 (1)	12 (22)^d^^,^ e	2 (4)	3.1
SMAD1		3 (3)	1 (1)	0.3								
SMAD2		0								1 (1)	1 (1)	0.8
SMAD3		2 (2)	1 (1)	0.3						1 (1)		
SMAD4		3 (3)^c^	2 (2)	0.6								
STK11		3 (4)^e^								1 (1)^b^^,^ e		
STK11IP		8 (11)^e^				2 (2)				3 (3)^e^		
TGFB1		1 (17)^e^				1 (3)				1 (5)		
TGFBR1		2 (2)	1 (1)	0.3						2 (2)	1 (1)	0.8
TGFBR2		5 (7)	3 (3)	0.9						1 (1)		
TP53		3 (3)	2 (2)	0.6		2 (2)	1 (1)	1.5		2 (2)	1 (1)	0.8
Total	1 (1)	223 (511)	59 (86)	25.8	2 (2)	64 (98)	11 (14)	20.6	2 (2)	116 (205)	21 (24)	18.6

REVEL > 0.5: REVEL score > 0.5.

# (#) Number of unique variants (number of individuals with one of the variants).

a Indicates that at least one carrier is homozygous recessive.

b At least one individual is homozygous recessive.

c Variant is present in African American samples.

d Variant is present in Asian samples.

e Variant is present in Admix samples.

*MLH1* and *MSH2* harbored the most predicted damaging variants (*n* = 16 and *n* = 15, respectively), while *CHEK2* had 13 and *APC* and *MSH3* both had 12 (Tables**** 3 and S2). Several genes had few predicted damaging variants, including those with one (*AXIN2*,* BUB1*,* CDKN1B*,* CDKN2A*,* SMAD2, TGFB1*, and *TGFBR1*), two (*AXIN1*,* BLM*,* BMPR1A*,* FLCN*,* GALNT12*,* PTEN*, and *SMAD3*), or three (*BMP4*,* SMAD1*, and *SMAD4*). No predicted damaging variants were found in *CTNNB1*,* NUDT1*,* PALB2*,* STK11,* or *STK11IP*.

The carrier rate of predicted damaging Tier 2 variants was highest in the young onset and unselected cases, followed by the pMMR cases (26%, 26%, and 21%, respectively) (Table 4). *MUTYH* had the highest percent carrier count of predicted damaging variants in three of the sample subsets (controls, FCCTX, and young onset), while in both the unselected and pMMR cases PMS1 had the highest percent carrier count. In the dMMR cases, RECQL5 had the highest percent carrier count and all of the cases with predicted damaging RECQL5 variants had loss of MLH1; however, the same variants were present in several FCCTX and young onset cases with normal tumor expression of MLH1. Thus, it is unlikely these variants are influencing the loss of MLH1. In the unselected cases, *MSH3* also had a high percent carrier count, primarily due to three variants present in five (p.Ser490Tyr and p.His827Arg) or four (p.Leu911Trp) individuals each (Table S2). Two of these variants (p.Ser490Tyr and p.His827Arg) were not present in Caucasian public control populations; however, they are present in African American controls in both 1000 Genomes and the Exome Sequencing Project (ESP), with frequencies ranging from 0.38 to 0.91% (Table S2). Indeed, of the 10 individuals with these two variants in our study, seven were classified as African American and three were of mixed descent. Several other genes had variants that were predominantly present in African American or admixed individuals, including *APC* (p.Ser26Arg), *CDKN2A* (p.Ala127Ser and p.Arg144Cys), *MLH3* (p.Asp1073Asn), and *PMS1* (p.Gly501Arg) (Table 3).

In FCCTX cases, *MUTYH* harbored the most unique variants (*n* = 6). Eight individuals had at least one pathogenic or suspected pathogenic *MUTYH* variant; no previous screening for common *MUTYH* mutations had been completed for these individuals. Three individuals harbored two *MUTYH* mutations, one homozygous for p.Pro405Leu, one homozygous for p.Gly396Asp, and one individual was a suspected compound heterozygote for p.Tyr179Cys and p.Pro359Thr, however, we could not determine whether the two variants were in *cis* or in *trans*. The remaining five individuals had a single *MUTYH* mutation. Eight individuals also harbored a *MSH2* variant (p.Gly322Asp) that met the random forest predicted damaging cutoff (0.536); however, this variant has been classified as not pathogenic by InSiGHT (Class 1).

In the unselected cases, *MSH2* harbored the most unique variants (*n* = 11) (Tables 4 and S2). Within this group, 15 cases harbored more than one predicted damaging variant in a single gene, including three individuals with two variants in *MUTYH* and three individuals with two variants in *PMS1* (Table 5).In the young onset cases, several genes had multiple predicted damaging Tier 2 variants, including *CHEK2*,* MLH1*,* MSH2*,* MSH3*,* MSH6*, and *MUTYH*. Three individuals in this subset had two heterozygous mutations in *MUTYH* and one individual had two heterozygous mutations in *MLH1*, although the phase of these alterations could not be determined (Table 5). In the pMMR cases, *PMS1* harbored the most predicted damaging variants, while in the dMMR cases *RECQL5* had the most individual with predicted damaging variants. No cases in either the pMMR or dMMR subsets harbored homozygous or compound heterozygous variants that were predicted to be damaging.

**Table 5:** Homozygous recessive and compound heterozygote cases

Individual	Sample Category	Gene	Variant 1	Variant 2
s_1204004008	Unselected	*APC*	Ser26Arg	Lys1436Glu
s_1207501457	Unselected	*APC*	Ser26Arg	Lys1436Glu
s_1208805000	Unselected	*CDH1*	Pro30Thr	Val55Gly
s_1204004011	Unselected	*CDKN2A*	His123Gln	Arg144Cys
s_1204003858	Unselected	*MLH1*	**c.589‐2A>G (splice)**	Val716Met
s_1204003974	Unselected	*MLH1*	**c.589‐2A>G (splice)**	Val716Met
s_1210304575	Young Onset	*MLH1*	**c.589‐2A>G (splice)**	Val716Met
s_1204003894	Unselected	*MSH3*	Gly896*	Leu911Trp
s_1204003966	Unselected	*MSH3*	Asn524Thr	Arg669Trp
s_1204003936	Unselected	*MSH6*	**Val717fs**	Glu1163Val
s_1210304546	FCCTX	*MUTYH*	**Tyr179Cys**	Pro359Thr
s_1217302838	FCCTX	*MUTYH*	**Pro405Leu**	**Pro405Leu**
s_1218802771	FCCTX	*MUTYH*	**Gly396Asp**	**Gly396Asp**
s_1204003854	Unselected	*MUTYH*	**Tyr179Cys**	Arg426Cys
s_1208805011	Unselected	*MUTYH*	**Tyr179Cys**	**Gly396Asp**
s_1208903273	Unselected	*MUTYH*	**Tyr179Cys**	**Gly396Asp**
s_1208704158	Young onset	*MUTYH*	**Tyr179Cys**	**Tyr179Cys**
s_1210304610	Young onset	*MUTYH*	**Gly396Asp**	**Gly396Asp**
s_1218101197	Young onset	*MUTYH*	**Tyr179Cys**	**Gly396Asp**
s_1218101281	Young onset	*MUTYH*	**Tyr179Cys**	**Ala357fs**
s_1218101344	Young onset	*MUTYH*	**Tyr179Cys**	**Tyr179Cys**
s_1204003928	Unselected	*PMS1*	Glu27Gln	Arg277*
s_1204003998	Unselected	*PMS1*	Gly501Arg	Gly501Arg
s_1208804993	Unselected	*PMS1*	Pro134Leu	Glu537Lys

Variants in bold represent Tier 1 variants or known pathogenic variants.

Seventeen unique in‐frame indel variants were identified in our samples, with 13 being present in a single case (Table S3). *MLH1* p.Lys618Ala (c.1852_1853delinsGC), *APC* p.Glu1157del, *AXIN2* p.His474_Ser475insHis, and *STK11IP* p.Ser739del were present in 13, seven, three, and two cases, respectively. Similar to what was seen with certain missense variants, the *APC* indel was present in African American and mixed decent cases, mirroring the higher frequency of this indel in individuals of African American descent in public control datasets. The majority of the indels were present in unselected cases (*n* = 12), likely due to the large sample size of the group. No in‐frame indels were present in the controls, while the pMMR and young onset cases each had two unique indels. Of note, the *MSH2* p.Asn596del indel present in one dMMR case is considered a Class 5 (Pathogenic) variant by InSiGHT, and the case harboring this variant demonstrated loss of MSH2 expression by IHC. The remaining in‐frame indels in the MMR genes (p.Leu94del and p.Ile217del in *MSH2* and p.Pro768del in *MSH6*) were not present in the InSiGHT database.

### Cases with multiple predicted damaging variants

In total, 117 samples (9.5%) harbored more than one Tier 1 variant, a pathogenic or likely pathogenic Tier 2 variant, or a predicted damaging Tier 2 variant. Two individuals had four predicted damaging variants, 22 had three predicted damaging variants, and 93 had two predicted damaging variants. In most individuals with more than one predicted damaging variant, the variants were present in different genes. However, a few cases had more than one Tier 1 or likely damaging Tier 2 variant in the same gene, as discussed above (Table 5). Six individuals were homozygous for predicted damaging mutations: five individuals for *MUTYH* and one individual for *PMS1*. Six additional individuals carried two heterozygous *MUTYH* mutations. Of the 11 individuals with two MUTYH mutations, nine had multiple polyps while there was no information available for the remaining two cases. Multiple heterozygous mutations were also detected *APC*,* CDH1*,* CDKN2A, MLH1, MSH3, MSH6,* and *PMS1*. Six of these individuals harbored a Tier 1 and predicted damaging Tier 2 variant. Additionally, three unselected cases harbored two Tier 1 variants in different genes. The first two harbored simple Tier 1 mutations: *MLH1* (p.Met35fs) and *MSH6* (p.Lys1358fs) in one individual, while another case harbored both *APC* (p.His2045fs) and *MSH3* (p.Gln74fs). As discussed previously, the third had large exonic deletions in both *MLH1* and *MSH2* (Table 3).

## Discussion

In this study, we sought to determine the scope and frequency of variants in 36 known or putative CRC susceptibility genes for five case subgroups (FCCTX, young onset, unselected, pMMR, and dMMR with no identified mutation) and an unaffected control group. We studied 18 genes known to be important in CRC susceptibility (*APC*,* AXIN2*,* BMP4*,* BMPR1A*,* CHEK2*,* MLH1*,* MLH3*,* MSH2*,* MSH3*,* MSH6*,* MUTYH*,* PMS1*,* PTEN*,* SMAD4*,* STK11*,* STK11IP*,* TGFBR2*, and *TP53*) and 18 genes suspected to play a role in CRC susceptibility (*AXIN1, BLM, BUB1, CDH1, CDKN1B, CDKN2A, CTNNB1, FLCN, GALNT12, NUDT1, OGG1, PALB2, REQL5, SMAD1, SMAD2, SMAD3, TGFB1,* and *TGFBR1)*. Overall, we identified 767 variants, including 29 nonsense, 43 frame‐shift indels, 13 splice site, six initiator codon, one stop‐codon variant, 658 missense, and 17 in‐frame indel variants in the 36 genes.

In total, we identified 72 of 1231 cases with pathogenic nonsense, frame‐shift, splice site, large deletions, or likely damaging missense variants in the MMR genes (5.8%), predominantly in the unselected and young onset subsets. Pathogenic mutations in *APC* were identified in 12 individuals (1.0%) and multiple pathogenic mutations in *MUTYH* were found in eight cases (0.6%). Overall, we identified pathogenic mutations in the MMR genes, *APC*, and *MUTYH* in 7.5% of our cases.

Large exonic deletions in the MMR genes, which can account for 15–45% of germline mutation in *MLH1* and *MSH2*, were identified in 1% of the cases (Baudhuin et al. 2005). In addition to the 1231 cases and 93 controls, 10 samples with known large deletions were also sequenced to determine if the deletions would be detectable. Eight of the ten deletions were identified; two smaller deletions were not identified, one in *MLH1* (~3 kb) and one in *MSH2* (~100 bp).

Tier 1 and predicted damaging Tier 2 variants were detected in all subsets for four genes (*MLH1*,* MSH6*,* MUTYH,* and *PMS1*). No Tier 1 or predicted damaging Tier 2 variants in *CTNNB1*,* PALB2*, or *STK11* were identified, while a single Tier 1 or damaging Tier 2 variant was detected in *AXIN2*,* CDKN1B*,* CDKN2A*, and *SMAD2*. Other genes for which a few Tier 1 or damaging Tier 2 variants include *GALNT12*,* NUDT1*,* PTEN*,* STK11IP*,* TGFB1*, and *TGFBR1*. While germline mutations in these genes may play a role in CRC pathogenesis, it is likely limited to very small proportion of cases. Including these genes in clinical testing panels would help identify the rare individuals with pathogenic mutations in these genes, however, it would also likely result in more VUS identified. Whether the difficulties in interpreting uncertain variants in relation to disease management and risk assessment are outweighed by the few clearly pathogenic variants identified will need further study.

In addition to the mutations found in cases, we also identified five Tier 1 variants present in control subjects. Mutations in two of the genes are responsible for autosomal dominant CRC and Loeys‐Dietz Syndrome (*MSH6* and *TGFBR1*, respectively). Results of all Tier 1 variants, regardless of case or control status, were reported to the site from where the affected individuals were recruited.

Eight genes had an additional 36 variants that would be present only in specific protein isoforms due to alternative splicing or alternative start sites, including *APC*,* CDKN2A*,* FLCN*,* MUTYH*,* OGG1*,* RECQL5*,* STK11*, and *TP53* (Table S4). For example, the *TP53* variant at chr17:7576541 is intronic in the predominant isoform (NM_000546.5); however, due to alternative splicing of exon 9, is considered a missense (p.Ser307Leu) in the gamma isoform (NM_001276695.1) of the protein. Several of these different isoforms of the genes have been found to be elevated in various types of cancer, but the impact on disease risk, progression, and outcome remains unclear.

While IHC testing can help determine which gene is implicated in the case of the MMR genes, we identified four cases with pathogenic nonsense or frame‐shift mutations in these genes despite tumor expression of the affected protein. Because previous IHC testing did not demonstrate loss of protein expression, germline sequencing of the MMR genes was not indicated. While not unheard of, the prevalence of intact MMR protein staining in conjunction with pathogenic mutations remains unclear. Previous studies have reported similar findings, especially in regards to *MSH6*; tumors in these cases may instead be phenocopies, have heterogeneous expression of the protein being tested, or the patient may have undergone neoadjuvant therapy (Radu et al. 2011; Shia et al. 2013). Additionally, IHC results are not always clearly positive or negative; centers may interpret the results differently, impacting the decision to proceed with germline testing. Thus, caution should be taken in regards to IHC testing results as they may not always accurately reflect the biology of the tumor. Additional studies are warranted to better understand how often this occurs and how it may lead to incorrect diagnosis for patients.

The ability to identify genomic variants via multigene sequencing panels has surpassed the ability to accurately classify variants in terms of functional importance. While rare nonsense, frame‐shift, and splice‐affecting variants are generally considered pathogenic when occurring in genes for which loss of function is known to be associated with disease, missense variants are much more difficult to assess. In silico programs, such as SIFT and PolyPhen, are often used, however, the pathogenicity predictions are not sufficiently reliable to use as stand‐alone evidence for pathogenicity and the programs are frequently not in agreement with one another; thus many missense variants remain classified as VUS. To aid in determining which variants were likely pathogenic, we used a new program, REVEL, utilizing a random forest score that incorporates multiple in silico prediction tools for use with rare variants (Ioannidis et al.). Of the 77 missense MMR variants in *MLH1*,* MSH2*, and *MSH6*, 53 had a REVEL score above our threshold of 0.5. Twenty of these were not present in InSiGHT; the remaining 33 were classified as Class 5 (pathogenic, *n* = 3), Class 4 (likely pathogenic, *n* = 1), Class 3 (uncertain, *n* = 17), Class 2 (likely not pathogenic, *n* = 6), or Class 1 (not pathogenic, *n* = 6). Using a more stringent threshold for the REVEL score would decrease the number of benign variants considered likely pathogenic, however, it may also decrease the chance of identifying true pathogenic variations.

Our study has several strengths. The large number of cases available through the Colon CFR allowed us to compare sample cases with varying characteristics. For example, while all case categories contained damaging or likely damaging variants in *MLH1*,* MSH6*,* MUTYH*, and *PMS1*, three genes (*CTNNB1*,* PALB2,* and *STK11*) had no damaging or likely damaging variants in any case subset and damaging variants in five genes were present in only a single case category (*AXIN2*,* CDKN1B*,* CDKN2A*,* NUDT1*, and *SMAD2*). Many of our cases had a family history of CRC, likely enriching for causative variants. Additional affected members may also be used in cosegregation studies to better predict the pathogenicity of rare missense variants.

Our study has some weaknesses. The modest number of controls limited our ability to compare frequencies of variants to those in our case subgroups. The majority of our variants were very rare or novel, present only in a single case. Sequencing additional controls, in conjunction with utilizing available public control databases and information from functional studies, will be essential to help establish which variants contribute to CRC predisposition. This study did not include all now known CRC susceptibility genes. *PMS2* was excluded because it is difficult to target due to the presence of multiple pseudogenes. *POLE, POLD1*, and *GREM1* were not included as they were identified as putative CRC susceptibility genes after completion of our custom capture design. Finally, while we were able to detect large deletions in eight samples with known deletions, we failed to find known deletions in two positive control samples. It is possible that coverage of the genes was not sufficient in these samples or that the deletions were difficult to detect due to their relatively small size. Additionally, verification of the identified deletions in cases has not yet been completed. Based on this, we must be careful in interpreting the data; there may be additional deletions not detected or some of the identified may be false positives. We were only able to search for large deletions in *MLH1*,* MSH2*, and *MSH6*, due to increased coverage of the regions, specifically designed into the targeted array. The remaining genes may also contain large deletions.

Yurgelun et al. completed a study similar to ours, in which they sequenced 1260 CRC cases with suspected Lynch Syndrome in a 25 gene panel (Yurgelun et al. 2015). There are several key differences in our study. First, we categorized cases by tumor MMR status, allowing comparison of case subsets. All of the cases in the Yurgelun et al. study were suspected Lynch Syndrome families, based on family history of Lynch Syndrome‐associated cancers. We also included >500 CRC cases with no prior IHC or MSI testing, reflecting what is more likely to be seen in the general population. Additionally, we sequenced 93 unaffected individuals, to help discriminate common, benign variants from those more likely to be truly involved in disease susceptibility. Of the 82 missense variants present in our controls, 50 were also present in cases. Only seven of these had predicted damaging REVEL scores, including one known pathogenic variant (*MUTYH*, Gly396Asp), one known benign variant (*MSH2*, Gly322Asp), and five variants of uncertain significance. The presence of these variants in controls at similar or higher frequencies than in cases favors a benign prediction.

In summary, we have utilized targeted sequencing to identify variants in the known and suspected CRC susceptibility genes. We identified multiple pathogenic and likely pathogenic mutations in our cases. Cases with discordant MMR tumor testing and sequencing results were discovered, perhaps due to tumor heterogeneity or phenocopies, underscoring that IHC and MSI testing is not always an accurate indicator of germline MMR status. As sequencing technologies improve and costs decrease, targeted sequencing of multiple CRC susceptibility genes is becoming an efficient method to screen individuals with suspected hereditary CRC, although the high frequencies of VUSs must be anticipated for a long time to come.

## Conflicts of Interest

The authors declare that they have no conflicts of interest to report.

## Supporting information


**Figure S1.** Missense variant classification.

**Table S1.** Individual Tier 1 variants.
**Table S2.** Individual Tier 2 variants.
**Table S3.** Individual in‐frame indels.
**Table S4.** Alternative splicing variants.
