# Supplementary figures and images for: Targeted sequencing of 36 known or putative colorectal cancer susceptibility genes
Source: Mol Genet Genomic Med. 2017 Jul 23;5(5):553–69. doi: 10.1002/mgg3.317 (PMC5606870; doi:10.1002/mgg3.317)

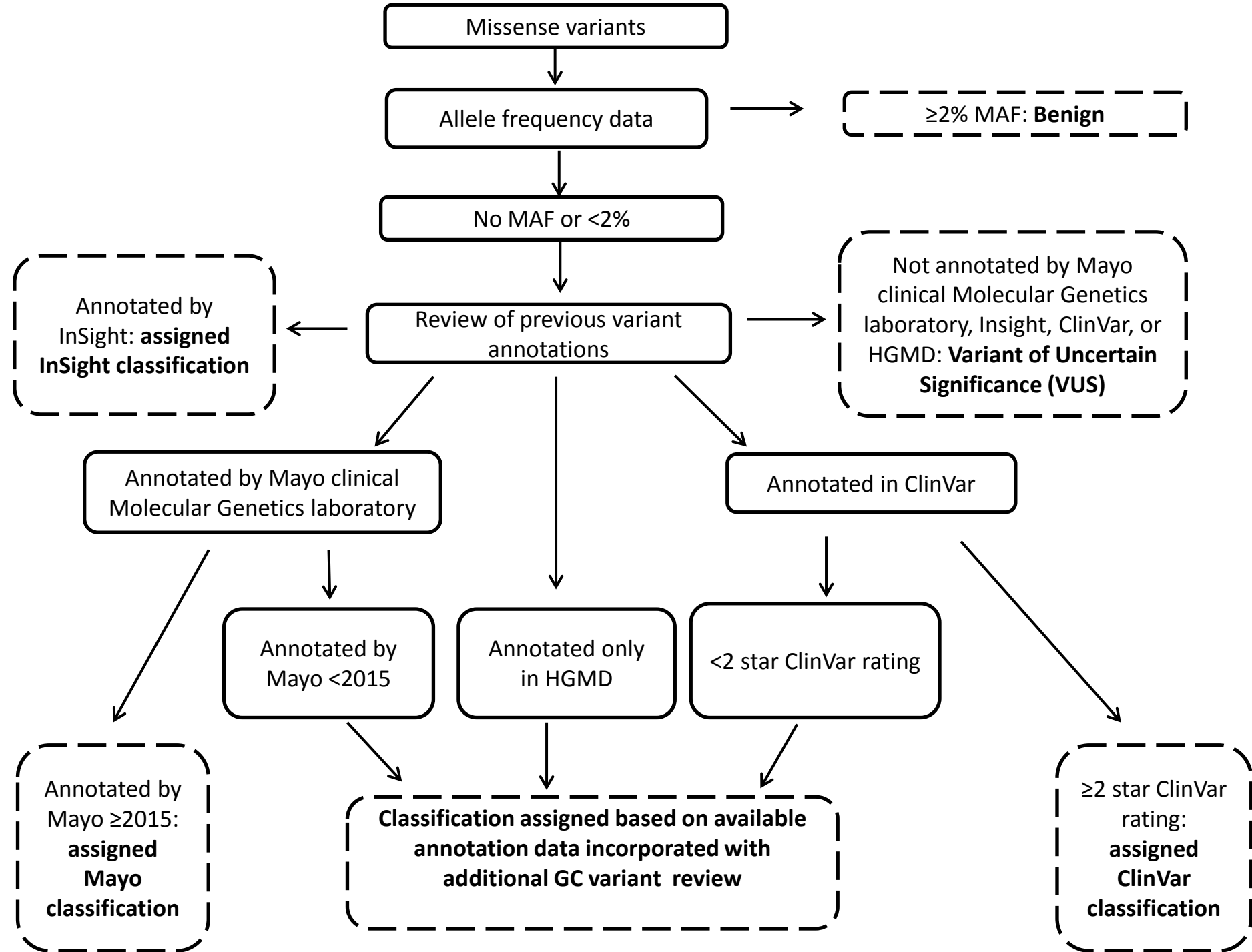

Supplement: Supplementary file 1 — Figure S1. Missense variant classification. [file MGG3-5-553-s001.pdf]
